# Public interest in America on cardiac arrest following cardiovascular events of Bronny and Damar: A Google trend study

**DOI:** 10.1016/j.ahjo.2024.100433

**Published:** 2024-07-30

**Authors:** Jasneel Kahlam, Alexander Sacher, John P. Reilly, David F. Lo

**Affiliations:** a Department of Internal Medicine, Stony Brook Southampton, Southampton, NY, USA; b Department of Internal Medicine, New York Presbyterian, Flushing, NY, USA; c Department of Cardiology, Stony Brook Southampton, Southampton, NY, USA; d American Preventive Screening & Education Association (APSEA), Stratford, NJ, USA

**Keywords:** Cardiovascular event, Cardiac arrest, Public interest, Google trends

## Abstract

**Background:**

Heart disease is one of the leading causes of death in the United States. Increased education and utilization of BLS by first responders have had a significant impact, but certain populations remain high risk, such as African Americans. Raising awareness among at-risk populations may lead to more bystander CPR performed, improving mortality rates. The influence of celebrity deaths and illnesses is an important driver of public awareness. Therefore, the cardiac arrests of both Bronny James and Damar Hamlin may have influenced cardiac arrest awareness.

**Methods:**

Google Trends data was pulled for the following search terms from 8/21/2022–8/14/2023: Cardiac arrest (disease), Cardiopulmonary Resuscitation (topic), Basic Life Support (topic), Myocardial Infarction (disease), Defibrillation (topic) and Automatic External Defibrillator (topic). The average relative search volume (RSV) for each search term was taken for a three-week period encompassing the week of and two weeks following the cardiac arrests of Damar Hamlin and Lebron James Jr., respectively. We used one-way ANOVA and independent sample *t*-tests to compare the average values of Damar Hamlin's and LeBron James Jr.'s incidents with their respective 12-month averages.

**Results:**

RSV was significantly higher surrounding Hamlin's cardiac arrest compared to James Jr.'s for Cardiopulmonary Resuscitation and Automatic External Defibrillator. RSV for Basic Life Support was increased in LeBron James Jr.'s time compared to the 12-month average and Damar Hamlin's incident. Compared to the 12-month average, Cardiac arrest, Cardiopulmonary Resuscitation, Defibrillation, and Automatic External Defibrillator during Hamlin's incident. Myocardial infarction RSV was higher during James Jr.'s incident compared to baseline. Over the long term, the search terms showed a significant increase after Damar Hamlin's incident when compared to before.

RSV was significantly higher surrounding Hamlin's cardiac arrest compared to James Jr.'s for “Cardiopulmonary Resuscitation” (23.56 vs. 22.0, *p* < 0.00) and “Automatic External Defibrillator” (19.59 vs. 19.4, p < 0.00). RSV for “Basic Life Support” was increased in LeBron James Jr.'s time compared to the 12-month average and Damar Hamlin's incident (80.9 vs. 66.88, *p* = 0.04). Compared to the 12-month average, “Cardiac arrest,” “Cardiopulmonary Resuscitation,” “Defibrillation,” and “Automatic External Defibrillator” during Hamlin's incident showed significant increases. “Myocardial infarction” RSV was higher during James Jr.'s incident compared to baseline (55 vs. 46.6, *p* = 0.026). Over the long term, the search terms showed a significant increase after Damar Hamlin's incident when compared to before (*p* < 0.05).

**Conclusions:**

Increases in the search terms for Hamlin's cardiac arrest compared to James Jr.'s cardiac arrest were associated with seeing the event live and increasing cardiac arrest awareness. Hamlins Cardiac Arrest also showed a significant increase in search terms over the long term. The increase in searches for “Basic Life Support” during James Jr.'s cardiac arrest indicates increased awareness. Also, the increase in myocardial infarction searches during both incidents could show confusion between cardiac arrest and myocardial infarction.

## Highlights

• Public interest in CPR increased in both the short- and long-term after Damar Hamlin's cardiac arrest.
• The public's interest in BLS increased in the short term during the cardiac arrest of Bronny James.
• There is a misunderstanding about the definitions of cardiac arrest and myocardial infarction.

## 1 Introduction

Cardiac arrest prevention is one of the most important topics in the field of cardiology. Throughout the United States, cardiopulmonary resuscitation (CPR) training is required for medical professionals. Although first responders, physicians, and nurses must master these skills, the most important time to initiate CPR is before the patient gets to the hospital. Therefore, our efforts to educate CPR must expand to the general public. It has been shown that bystander CPR, both dispatch-assisted and self-led, improved neurological outcomes when compared to no bystander CPR in out-of-hospital CPR [1,2,3,4]. Additionally, bystander CPR, with and without dispatcher assistance, showed improvement in achieving ROSC and survival at admission and at 30 days [5,6]. Over a longer period, bystander CPR was able to increase 5-year survival time while decreasing the total cost of hospitalization [7].

Despite the demonstrated importance of bystander CPR, there has yet to be much success in getting bystanders to learn CPR. A survey done in Australia showed that despite the public's knowledge of CPR's existence, the largest barrier to training the public was “never thinking about it.” [1]. One of the ways that this lack of awareness can be fixed is through increased public attention, namely through celebrities [8]. Another common barrier to bystanders learning CPR is a lack of confidence in performing the procedure [9]. However, studies done to assess confidence in CPR for non-healthcare showed that confidence and willingness to do CPR increased after taking a BLS course [10,11].

Celebrities have a strong influence on what we focus on, especially regarding medicine. For instance, public figures who are diagnosed with certain diseases promote education and conversation about these diseases. After basketball player Earvin “Magic” Johnson disclosed his diagnosis of HIV, there was an increase in concern and interest in AIDS education among urban males [13]. In the face of actor Chadwick Boseman's untimely death, there was an increase in the Google search volume for colorectal cancer [14,15]. Not only has public interest shown an increase, but celebrity influence also inspires patients to take more action.

During the time that Katie Couric, a former journalist for ABC, launched her colon cancer campaign, there was a significant increase in colonoscopies per month [16]. Even though these celebrity events have had positive effects on healthcare, celebrities can also have a negative influence. It has been shown that nominees for the Teen Choice Awards between 2000 and 2014 were more likely to sponsor sugary, unhealthy foods and beverages [12]. With the recent cardiac arrests of LeBron James Jr., “Bronny,” a college basketball player and son of hall-of-famer LeBron James, and professional football player Damar Hamlin, we aim to see if there is an opportunity to increase public awareness of cardiac arrest.

Damar Hamlin is a 25-year-old African-American male who is a professional American football player for the Buffalo Bills. During a Sunday night football game against the Cincinnati Bengals, Damar was struck in the chest during a routine tackle and was unresponsive. Medical responders immediately administered CPR and were able to revive Damar twice before reaching Buffalo General Hospital. After being diagnosed with commotio cordis, Damar was eventually released a week later from the hospital.

Lebron James Jr., also known as Bronny James, is an 18-year-old male who is a college basketball player at the University of Southern California. While practicing at his college facilities on July 24th, 2023, Bronny suffered a cardiac arrest. Bronny James was sent to Cedar Sinai Hospital and was discharged home three days later. Unfortunately, not much is known about the resuscitation efforts since the incident happened outside of the public eye.

Previously, there was one study that assessed the changes in Google Trends (GT) during Damar Hamlin's cardiac arrest [17]. In their study, they showed a significant increase in the following search terms: “CPR”, “Cardiopulmonary Resuscitation”, and “commotio cordis”, during the 5 days after Damar Hamlin's incident. Although these results are promising, this study did not assess any long-term changes or effects on search terms related to BLS or defibrillation. At the time of its publication, LeBron James Jr.'s cardiac arrest did not happen, so there were no other events to compare Damar's event to. To date, there have been no studies assessing GT for cardiopulmonary resuscitation-related terms during the time of Lebron James Jr.'s cardiac arrest. Our study reinforces the impact of Damar Hamlin's cardiac arrest on both short- and long-term interest in BLS and demonstrates Bronny's influence on it as well.

## 2 Method

A retrospective observational study was performed using data from Google Trends (GT) (Google, Mountain View, California) on October 23, 2023. GT is a publicly available dataset that assesses relative search volumes (RSV) for a certain region and timeframe. GT was used as it is the most popular search engine that allows for comparing different timeframes and multiple search terms. Importantly, it offers a good way to see how public interest changes with current events. GT data is quantified by RSV, which ranges from 0 to 100, indicating how often a term is searched within a specific timeframe. In this study, the terms “Cardiac arrest,” “Cardiopulmonary Resuscitation,” “Basic Life Support,” “Myocardial Infarction,” “Defibrillation,” and “Automated External Defibrillator” were assessed for two 12-month periods. Data was recorded by week. The first period was assessed from 8/21/2022 until 8/14/2023. The rationale for choosing the 12-month period from August 21, 2022, to August 14, 2023, was to provide a comprehensive view of search trends over a substantial time frame surrounding both Damar Hamlin and LeBron James Jr.'s cardiac arrest events. This duration encompassed a full year, allowing for a robust comparison of search volumes and patterns. While selecting a ± 6-month period around each event could have provided a more immediate comparison, it might not have captured seasonal variations or longer-term trends adequately. By utilizing the same 12-month average for both events, despite their occurrence in different months, a standardized baseline for comparison, minimizing the impact of seasonal fluctuations and ensuring a consistent evaluation framework. This approach enhances the validity of the comparison by establishing a common reference point for assessing changes in search behavior across different time periods. Additionally, if a +/− 6 month was placed around both events, then the baseline would have been smaller for Bronny because the beginning of the 6 month period would have included some of the Damar Hamlin effect. The weekly RSV values were averaged for each term and compared using a one-way analysis of variance (ANOVA). RSV was used to quantify google search changes as Absolute volume search was not available as an extension on google trends studies. Relative search volume, despite failing to compare exact number to exact number, allowed us to develop a point of comparison for changes in searches across the one year baseline.

Next, three-week intervals encompassing the week of and two weeks following Damar Hamlin's (1/2/23) and LeBron James Jr.'s (7/24/23) cardiac arrests were evaluated. This baseline RSV was compared to the RSV surrounding Lebron James Jr.'s and Damar Hamlin's cardiac arrests. Then, the averaged RSV of Hamlin's and James Jr.'s cardiac arrests were compared to each other. The *p*-values are derived from independent sample *t*-tests comparing RSV for each search term between the two cardiac arrest events. The initial 12-month period RSV for each search term was set as the baseline.

Additionally, the long-term effects of Damar Hamlin were analyzed. The search volumes were assessed for each of the search terms above from 9/1/2022–12/25/2022 (*n* = 17) and from 1/22/2023–10/1/2023 (*n* = 34). This was done to assess for the year period surrounding the Damar Hamlin incident while eliminating the 3-week period surrounding LeBron James Jr. (7/23–8/12). This was done to eliminate any RSV changes related to the Bronny James incident. This length of time was chosen because most studies that assessing a change in Google trends due to a celebrity event showed that events only spurred changes for a couple of weeks [14,26]. From this, it was decided to assess less time before and more time after the Damar Hamlin event to further emphasize the long term effect. Although we could have split this one year period 26 weeks before and 26 weeks after, splitting it 17 and 34 allowed us to make assess changes for a longer period of time without affecting the baseline. The last two columns show comparisons of the three weeks around each event compared to the 12-month average. All statistical analysis was performed using SPSS version 24 (IBM Inc., Armonk, New York) (Table 1).

**Table 1:** List of search terms used.

Search term	Alternate terms
Cardiac arrest	Heart stoppage
Cardiopulmonary resuscitation	CPR, Cardiopulmonary revival
Basic life support	BLS
Myocardial Infarction	MI, Heart attack, coronary occlusion, cardiac ischemia
Defibrillation	Defib, Cardioversion
Automated external defibrillator	AED, Automated defibrillator, electronic resuscitation device

## 3 Results

### 3.1 Long-term effects of Damar Hamlin's cardiac arrest

As shown in Table 2, there was a significant increase in RSV after the event from 3.82 to 4.47 (*p* = 0.034) when comparing the average RSV for “Cardiac Arrest” before and after Hamlin's cardiac arrest. For the search term “Cardiopulmonary Resuscitation,” there was an increase in RSV from 18.59 to 23.56 (*p* < 0.00) after Hamlin's cardiac arrest. For the search term “Basic Life Support,” the RSV increased from 66.88 to 86.15 (p < 0.00) after Hamlin's cardiac arrest. For the search term “Myocardial infarction,” there was an increase in RSV from 42.94 to 45.74 after Hamlin's cardiac arrest (*p* = 0.026). For the search term “Defibrillation,” there was an increase from 33.18 to 35.88 (*p* = 0.022) after Hamlin's cardiac arrest. For the search term “Automated External Defibrillator,” there was an increase in RSV from 14.18 to 19.59 (*p* < 0.00) during Hamlin's cardiac arrest.

**Table 2:** Mean and standard deviation for RSV of each search term in the 17 weeks before and 34 weeks after the cardiac arrest of Damar Hamlin.

	Average RSV (SD)		P-value
	Before DH arrest (n = 17 weeks)	After DH arrest (n = 34 weeks)	
Cardiac arrest	3.82 (0.883)	4.47 (1.05)	0.034
Cardiopulmonary resuscitation	18.59 (2.12)	23.56 (2.31)	<0.00
Basic life support	66.88 (7.70)	86.15 (6.61)	<0.00
Myocardial infarction	42.94 (3.07)	45.74 (5.61)	0.026
Defibrillation	33.18 (3.76)	35.88 (3.89)	0.022
Automated external defibrillator	14.18 (2.19)	19.59 (2.46)	<0.00

Abbreviations: RSV: Relative search volume; DH: Damar Hamlin.

### 3.2 RSV between LeBron James Jr. vs. baseline

As shown in Table 3, when comparing the RSV for the search term “Cardiac Arrest” between LeBron James Jr. and the 12-month baseline, there was an increase in RSV from 8.02 to 22.7 (*p* = 0.129) around the time period of LeBron James Jr.'s cardiac arrest. For the search term “Cardiopulmonary Resuscitation,” there was no significant difference in RSV during the time of LeBron James Jr. when compared to the 12-month baseline (22.0 vs. 23.2, respectively, *p* = 0.857). For the search term “Basic Life Support,” there was a significant increase during James Jr.'s cardiac arrest from 94.3 to 80.9 (*p* = 0.04) when compared to the 12-month average. For the search term “Myocardial Infarction,” there was no significant difference in RSV around the time of James Jr.'s cardiac arrest when compared to the 12-month average (55 vs. 46.6, *p* = 0.251). For the search term “Defibrillation,” there was no significant difference in RSV around the time of James Jr.'s cardiac arrest when compared to the 12-month baseline (36.3 vs. 36.8 respectively, *p* = 0.944). For the search term “Automated External Defibrillator,” there was no significant difference between the RSV during the James Jr. cardiac arrest and the 12-month average (19.3 vs. 19.4, *p* = 0.992).

**Table 3:** One-way ANOVA of each search term in the three weeks surrounding the cardiac arrests of Damar Hamlin and Lebron James Jr., as well as the 12-month average RSV.

	DH Mean (SD)	LJJ Mean (SD)	DH vs. LJJ (*p*-value)	12-month total Mean (SD)	DH vs. 12-month Avg (p-value)	LJJ vs. 12-month Avg (p-value)
Cardiac arrest	48.3 (46.2)	22.7 (28.0)	0.38	8.02 (15.3)	0.269	0.129
CPR	54.7 (39.7)	22.0 (0)	0.034	23.2 (11.5)	0.304	0.857
BLS	87 (4.6)	94.3 (1.5)	0.031	80.9 (11.9)	0.388	0.00
MI	76.7 (21.4)	55 (26.1)	0.71	46.6 (11.3)	0.00	0.251
Defibrillation	64.3 (31.8)	36.3 (3.5)	0.062	36.8 (10.1)	0.271	0.944
AED	51.3 (42.4)	19.3 (1.52)	0.038	19.4 (12.0)	0.322	0.992

Abbreviations: DH: Damar Hamlin; LJJ: Lebron James Jr.; Avg: Average; CPR: Cardiopulmonary Resuscitation; AED: Automated External Defibrillator; MI: Myocardial Infarction; BLS: Basic Life Support.

### 3.3 RSV of Damar Hamlin vs. baseline

When comparing the RSV for the search term “Cardiac Arrest” and “Cardiopulmonary Resuscitation” between Damar Hamlin and the 12-month average, there was no significant difference (48.3 vs. 8.02, *p* = 0.269, 54.7 vs. 23.2, *p* = 0.304). For the search term “Basic Life Support,” there was no significant difference in RSV between Hamlin's cardiac arrest and the 12-month baseline (87 vs. 80.9, *p* = 0.388). For “Myocardial Infarction,” the time period during Hamlin's cardiac arrest showed a significant increase in RSV when compared to the baseline (76.7 vs. 46.6, *p* = 0.00). For the search term “Defibrillation,” there was no significant difference in RSV between the Hamlin cardiac arrest and the 12-month average (64.3 vs. 36.8, *p* = 0.944). For “ Automated External Defibrillator,” there was no significant difference between Damar Hamlin's cardiac arrest and the 12-month average (51.3 vs. 19.4, *p* = 0.322). All results are shown in Table 3.

### 3.4 RSV of Damar Hamlin to LeBron James Jr

When comparing the RSV for the term “Cardiac Arrest” between Damar Hamlin's cardiac arrest and LeBron James Jr.'s cardiac arrest, there was no significant difference in searches (48.3 +/− 46.2 vs. 22.7 +/− 28.0 (*p* = 0.38). When comparing the RSV for the term “Cardiopulmonary Resuscitation,” the time frame around Damar Hamlin's cardiac arrest had a significant increase in search volume when compared to the LeBron James Jr. time frame (54.7 vs. 22.0, *p* = 0.034). When comparing the RSV for the search term “Basic Life Support,” the Lebron James Jr. timeframe had a significant increase in search volume compared to the Damar Hamlin timeframe (94.3 vs. 87, *p* = 0.031). When comparing the RSV for the search term “Myocardial infarction,” there was no significant increase in search volumes between the two groups (76.7 vs. 55, *p* = 0.71). When comparing the RSV for “Defibrillation,” there was no significant difference in search volumes between the two groups (64.3 vs. 36.3, *p* = 0.062). When comparing the RSV for “Automated External Defibrillator.” The Damar Hamlin time frame showed a significant increase in search volume when compared to the LeBron James Jr. time frame (51.3 vs. 19.3, *p* = 0.038). All results are shown in Table 3.

### 3.5 Google search trends

**Fig. 1:**
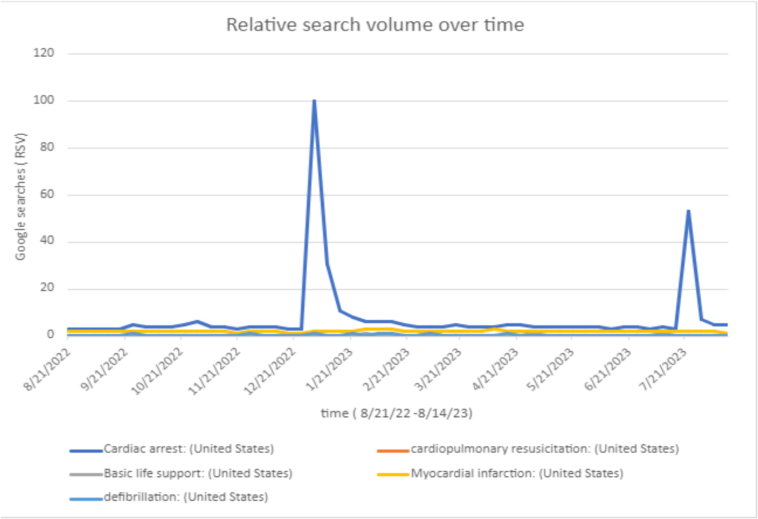
Line graph of the Google search trends.

As shown in Fig. 1, search results for Cardiac arrest peaked during the week of 1/2/23–1/9/23 and peaked again, July 23–292,023, corresponding with cardiac arrest events (blue line). Other lines that were included were for Myocardial infarction(yellow), Basic life support (gray), Cardiopulmonary resuscitation (red), defibrillation (light blue).

## 4 Discussion

### 4.1 Clinical implications

This study aimed to assess whether the cardiac arrests of LeBron James Jr. and Damar Hamlin influenced the public interest in cardiac arrest. One significant finding from this study is that Damar Hamlin's cardiac arrest showed a long-term change in the baseline for all the search terms involved. This may have been because his cardiac arrest was live on Sunday Night Football in a late-season regular season game. Viewers were able to see CPR and AED live, which may have left a more lasting impact on viewers. This was different from the Bronny James incident, which was behind closed doors. These long-term changes could serve as an opportunity to further educate the public about cardiac arrest and convince bystanders to learn CPR. During the time after his cardiac arrest, Damar Hamlin made efforts to help with CPR training by offering free courses and giving away AEDs to fans around the Buffalo Bills home stadium. These actions may have played a role in the long-term increase in cardiac arrest searches and possibly increasing CPR course enrollment among Americans, particularly the African-American population.

Increased enrollment in these courses may lead to increased performance in CPR, as it has been shown that one of the largest barriers to CPR is a lack of confidence in skill [18]. Also, it is reasonable to conclude that the period of time after Damar Hamlin's cardiac arrest was the most publicized event/topic in regards to CPR and cardiac arrest. It was not until Bronny James Jr. that cardiac arrest/CPR were publicized on a national scale, and that three week period was eliminated from long term effects of Damar Hamlin's event. Additionally, not all public news correlates with increased interest. For example, there was an increase in Twitter activity about Christian Danneman Eriksen's cardiac arrest during the EURO 2020 championship match. Despite this increased public outpouring, when Eriksen's cardiac arrest was analyzed for this study, the search volume was irrelevant and excluded from the study.

### 4.2 Public awareness and celebrity influence

Given the Damar Hamlin effect mentioned above and the increase in BLS searches during the time of Bronny James' cardiac arrest compared to baseline, this may reinforce the notion that celebrities have a huge influence on where our attention is shifted. After seeing a public figure who was stricken with cardiac arrest, the public may have become more aware and interested in searching cardiac arrest and BLS-related terms. This could be attributed to herd behavior or the notion that observing Damar's focus on CPR education may have influenced their thinking [19]. The fact that both of these athletes were African Americans might help bolster interest in cardiac arrest in their communities, which might help decrease the high cardiac mortality rate in their communities if they can initiate CPR earlier.

Additionally, there were increases in myocardial infarction during James Jr.'s and Hamlin's cardiac arrest compared to the baseline, with a significant increase during Hamlin's cardiac arrest. This may indicate that the public may think that myocardial infarction and cardiac arrest refer to the same phenomenon. This is especially important to note because neither Bronny nor Damar had a cardiac arrest due to coronary artery disease but by commotio cordis and a congenital heart defect, respectively. In a study done in London, half of the interviewees responded that they did not know the difference between cardiac arrest and myocardial infarction, and of those who did had trouble explaining the difference [20], Additionally, a study that analyzed newspaper articles involving cardiac arrest showed that cardiac arrest was only correctly and clearly identified in the title in 54 % of the articles [24]. Further education about this difference is crucial because it will eliminate the notion that healthy individuals, such as athletes, cannot go into cardiac arrest because they do not have heart disease.

While this study demonstrates potential for CPR education, some articles argue that the media may over glorify it. Fields et al. argue that the newspapers illustrate a more positive image of cardiac arrest survival compared to regional epidemiological data, the epidemiological data does not account for whether quality CPR was performed or the time to initiation of CPR [21]. One article criticizes the phenomenon of “Cough CPR,” where a patient is instructed to cough when feeling symptoms of a heart attack [23]. However, this article does not present any data supporting the misinterpretation of CPR by social media. Additionally, it argues that cough CPR has had some fluctuations in its Google trends over a 12-month period, but fails to analyze any data supporting this claim.

### 4.3 Further directions

There is still a need for a clinical study to assess the impact of this increased awareness of cardiac arrest. Future studies can assess whether these cardiac arrests played a role in increasing enrollment in bystander CPR courses. There is also a need to assess whether these increased searches lead to a higher rate of bystander CPR being performed on out-of-hospital cardiac arrest patients. These studies are important to explore as they will assess whether or not this increase in interest is spiking action to get trained and perform CPR when needed.

Additionally, with the rise of Artificial intelligence (AI) as a source for information, it is important to assess how it plays a role providing accurate and awareness of cardiac events. One recent study, Scquizzto et al. 2024, showed that when asking a list of 40 questions that were created by the Sudden Cardiac arrest UK committee to ChatGPT, the questions were highly rated for clarity, relevance, accuracy, and comprehensiveness [24].

### 4.4 Limitations

In this study, there are some important limitations that we need to assess. From this study, it is unclear whether these Google searches lead to increased education in BLS. People may have searched the terms out of curiosity about what happened to the athlete as opposed to an effort to learn the information. Additionally, some of the terms may be too advanced for the general public to associate with. For instance, most people are familiar with the acronym “CPR” as opposed to “Cardiopulmonary Resuscitation.” Although these terms have the same meaning, the public might not be aware that CPR stands for cardiopulmonary resuscitation. There were only six search terms used in this study, which does not account for the infinite number of terms that could have been searched. Additionally, there could have been misspellings or reordering of some of the search terms included in this study.

Another potential limitation could arise from concurrent news or events that might have influenced heightened interest in cardiac arrests, serving as confounding factors. However, upon conducting a search during those periods, no major news events in regards to Cardiac arrest were found. Additionally, around this time, Damar Hamlin was doing community outreach for CPR education, which would support the idea that these celebrity events could be used to educate the public. Finally, the limited observation of the time assessed could have limited the data. For instance, the long-term effects of Damar Hamlin's cardiac arrest were assessed only over a 34-week period. Further studies would be needed to assess how long this spike in cardiac arrest interest will last and if Bronny James' cardiac arrest would have a similar long-term impact.

## 5 Conclusion

This study explains both short-term and long-term increases in interest and awareness regarding cardiac arrest during and after Damar Hamlin's cardiac arrest on January 2nd, 2023. Additionally, there was a short surge in interest in BLS during the period surrounding LeBron James Jr.'s cardiac arrest on July 24th, 2023. Our paper highlights a misunderstanding among the public regarding the distinction between myocardial infarction and cardiac arrest as well. These findings emphasize the need for continued efforts to educate the public about these critical medical events and the importance of bystander intervention through CPR training. Future research should explore whether these heightened levels of interest translate into increased enrollment in CPR courses and improved outcomes for out-of-hospital cardiac arrest patients. Despite the observed limitations, this study contributes valuable insights into the public response to high-profile cardiac arrest events and underscores the ongoing importance of public health education in this area.

## Funding

No funding was received for this study/paper.

## CRediT authorship contribution statement

**Jasneel Kahlam:** Writing – review & editing, Writing – original draft, Supervision, Project administration, Methodology, Investigation, Formal analysis, Data curation. **Alexander Sacher:** Methodology, Data curation. **John P. Reilly:** Supervision, Investigation. **David F. Lo:** Writing – review & editing.

## Declaration of competing interest

The authors declare that they have no competing interests.
